# On the Puerperal Fever, and an Explanation of the Mode of Treating It

**Published:** 1802-11-01

**Authors:** 

**Affiliations:** Harbourg, in the Electorate of Hanover


### *On the Puerperal Fever, and an Explanation of the Mode of treating it; by* Dr. MICHAELIS, *of Harbourg, in the Electorate of Hanover.*

THE Puerperal Fever has always been confidered as a very fatal diforder incident to pregnant women; but I had the good fortune in my practice, to hit upon a method of treating it with a tolerable degree of fuccefs, even when complicated with lacteal metaftafes upon the lungs. As the modes of treatment which I have adopted, differ from thofe that have ufually been followed, it may perhaps be acceptable to practitioners to detail the particulars of it.

It is well known, that this diforder, to which all women are fubject, fooner or later, after their delivery, generally begins with cold fits of various degrees of violence, which are fucceeded by heat and perfpiration; the heat and febrile motions gradually fubfide during and after the perfpiration, though without entirely ceafing, and the cold fit and heat return at uncertain intervals, frequently on the fame day. The pulfe is irregular, feldom full, generally fmall, quick, intermittent, and altogether is what may be called a nervous pulfe. The patient foon after perceives a painful fenfation in fome parts of the body, commonly in the belly, which fwells, or in the cheft with an oppreffion of breathing; fometimes the limbs begin to fwell, particularly the legs, and the tumour is either eryfipelatous or moftly pale, refembling an œdema, except its having a great degree of heat. The breafts now become more flaccid, the milk diminifhes or difappears entirely, or if the breafts had not already been filled, when the diforder has taken place, little or no milk will enter them. The lochiae are fometimes fuppreffed, at other times flow irregularly, and fometimes they are not difturbed at all. The patient at firft is frequently thrown into a gentle delirium, which only, in fome cafes, rifes to a greater degree of violence, namely, when the head is particularly affected. Then it alfo happens, that the organs of the fenfes fuffer in various ways; for inftance, by blindnefs, with a dilatation of the pupil, difficulty of hearing, &c.

If the abdomen be principally attacked, either vomiting or diarrhœa of a yellowifh watery matter fupervenes, in which milky particles are contained, or a coftivenefs takes place, but in either cafe it becomes tumid and extremely painful; the more it fwells, the more the oppreffion is increafed; the diarrhœa leffens the intumefcence of the abdomen, but frequently it is augmented even during the moft copious diarrhœa, and continues to to the moment of death. A diarrhœa always makes the diforder worfe, when it is too violent, nor can it ever be deemed falutary, unlefs it has the effect of diminifhing the fwelling of the belly, and of affording relief.

If the cheft be chiefly affected, an oppreffion of the breath attended with ftitches in either of the fides, which fometimes fhift about; a cough, a four fmelling breath, juft like four milk, and a white expectoration retaining the fame fmell, will be obferved. There are likewife inftances, when the pharynx is affected; then, befides anginous fymptoms, thofe parts are covered with a white fourifh mucus fubftance. Any part peculiarly affected fwells, becomes extremely painful, fometimes breaks, and there iffues a milky or purulent matter, which in the manner of milk fometimes is feparated into cafeous, watery, and oily components. Sometimes the fwelling fubfides without breaking, but very flowly; at other times it neither fubfides nor is collected into matter in any one fpot, but becomes œdematous, which generally ends fatally.

Thefe fymptoms, of which frequently feveral are combined together, are accompanied with profufe fweating, which produces no benefit, but caufes languor, and is difcernible by a four fmell. The urine is thick and troubled, and forms a thick fediment. The limbs are moftly cold. Sometimes a miliary eruption fhows itfelf on the whole body or on the cheft, which however is merely accidental, and changes not in the courfe of the diforder. The face of the patient is generally pale, and looks emaciated, except at the time of the hot paroxyfm; often they have a great inclination to fleep. The difeafe is obferved to recur, though feemingly completely cured, without any evident caufe; and even when all the perilous fymptoms are removed, the pulfe will for a long time after remain febrile; the belly fwoln and fometimes painful; and before the pulfe is become entirely quiet, the difeafe is always apt to return.

In thofe cafes, which I had an opportunity of feeing in the Vienna Lying-in Hofpital, the patients were in general fuddenly carried off, after having been afflicted with great anxiety and delirium, attended with an extraordinary fwelling of the belly, violent pains in the breaft, and great oppreffion of breath, and at laft the limbs grew cold, as in a real gangrene. In thefe cafes the fymptoms were, as far as I know, not increafed by a hot treatment or other improper remedies. In feveral inftances, when the fymptoms appeared to be gentle, and the conftitution afforded good hopes, death neverthelefs followed. Among thofe few of my patients who died in confequence of this malady, there was one, in whom the diforder at firft feemed fo infignificant, that I was induced to retard the application of that remedy, medy, of whose superior utility experience has since so often convinced me.

At the dissections which I witnessed, especially in the Vienna Lying-in Hospital, there was always found in the belly, if that happened to be the part affected, a green yellowish matter, which was incrusted about the solid parts, particularly the bowels and the uterus, and which from the state of fluidity and the appearance of serum lactis, passed over into the consistency of a tough jelly; the smell of this matter was either sour like that of milk, or it stunk like rotten cheese. The bowels immediately underneath it were in general whiter than natural, and it was but seldom that you might observe in some spot or other, veins highly filled with blood or some pale redness, which however, could hardly be called an inflammation. I frequently perceived this appearance at one or the other of the tubæ Fallopii; the uterus had generally its natural shape, though it was at times inflamed and gangrenous. Sometimes, especially in those cases where a flakey sort of milk was evacuated by the stools, the intestines were found to be filled with similar matter.

Of those who died with affections of the breast and milky expectorations, I had no opportunity of opening any one; but I think myself justified in supposing, from the circumstance of matter like milk being thrown out, which was easily to be distinguished from common phlegm by its colour, smell, and taste; that matter of the like kind with that in the belly must be found in the lungs and in the cavity of the thorax, especially as these patients generally die by attacks of suffocation.

Nor does it appear to me improbable, that even in the brain such milky effusions may be met with, when symptoms of stupor, blindness, and similar affections, which lead us to suppose a pressure of the brain, precede dissolution. I generally saw several women taken in at once, and always when the constitution was rheumatic or catarrhous, which for some years past has been here the prevailing constitution. It often attacked those who being advanced above thirty years, lay in for the first time. Frequently difficult deliveries, a sedentary life during the state of pregnancy, the catching of cold, excess in diet, passion, imprudent stoppage of the milk in those who either could not or would not suckle, violent hæmorrhagies, or incautious purgings preceded, as debilitating causes. Sometimes the shivering was antecedent to the delivery, and the woman laboured under a rheumatic fever before she was brought to bed.

In a young healthy person, who was pregnant for the first time, violent pains in the knee were felt the day before her delivery, which I indeed lessened, but they returned with increased force after the delivery, (not a very difficult one, though though the forceps in the laſt ſtage was neceſſarily had recourſe to). The knee continued to ſwell, and ſeemed to contain a fluid; no milk appeared in the breaſts; and it was not till after a conſiderable time, that I ſucceeded in reducing the tumour, without cauſing it to break. But even then no milk returned into the breaſts. In this ſhape, and under theſe circumſtances, I obſerved the Puerperal Fever, concerning the nature of which, ſo many different and contradictory opinions continue to prevail.

Without mentioning the ſingular ideas, which many phyſicians have entertained on this ſubject, being ſeduced by ſome inſulated phenomena to form general concluſions, and without entering into a uſeleſs refutation of the ſame, (ſince diſſections do but prove too clearly that neither the inflammation of the uterus, of the omentum, of the peritonæum, nor of other inteſtines, are always to be met with, and ſince there are Puerperal Fevers, in which the abdomen ſuffers not at all, or but very little and tranſitorily) I ſhall only touch upon a generally prevailing opinion, according to which this diſeaſe is nothing but what is commonly called a gaſtric fever, attended with nervous ſymptoms, which however, may not unfrequently be complicated with a putrid and inflammatory character; and further, that lacteal metaſtaſes, as well as inflammation of the uterus, are but accidental ſymptoms of the diſeaſe, and not eſſentially connected with it. Thoſe phyſicians may juſtly be allowed to define or characterize that as a fever, in pregnant women, to which, on account of the nauſea and vomiting, foul taſte and tongue, they aſſign the name of gaſtric, and to treat it with evacuants; but they have no reaſon to conclude that the diſeaſe which I have deſcribed, and which among medical writers bears the appellation of Puerperal Fever, is to be comprehended under that fever, as it is accompanied with ſymptoms that widely differ from thoſe which are to be obſerved in gaſtric fevers.

With this diſorder of lying-in women, lacteal metaſtaſes are eſſentially connected, being obſervable in different ſpots and different degrees, either aſſuming the appearance of milk or of a coagulable lymph; ſometimes accumulated in one place; at others diffuſed through whole limbs; and alſo attended either with a total want of milk in the breaſt, or with a conſiderable diminution of it. If it be maintained, as has been done by ſome, that this matter is no milky ſubſtance, which already had been ſecreted in the breaſt as milk, or would have undergone that proceſs, having already the reſemblance of milk; and if it be attempted to prove this notion by the quantity of that matter found in the belly, it will be refuted by the chemical experiments which Selle and Hermbſtaedt have made with this ſubſtance.

As

As for the quantity of that fluid, it need only be confidered, that the portion of milk, which during feveral days and weeks is ufually fecreted in the breaft of a lying-in perfon, is, to all intents and purpofes, as great as the former, and frequently even furpaffes it. The poffibility of the milk being received into the lymphatic veffels, and being depofited in other parts, or of the procefs of fecretion being obftructed, ftands in need of no proof or argument. But it will appear indubitable, from diffections, that the fymptoms in puerperal fever, efpecially thofe that occur in its ufual fhape, fuch as intumefcence of the belly, do not proceed from bilious impurities, nor can be referred to the common appearances attendant on gaftric fevers.

There are fome phyficians who coincide with this opinion; yet contend, that though lacteal metaftafes take place in puerperal fevers, the caufe of fuch metaftafes, neverthelefs, muft be a gaftric ftimulus. In fupport of their ideas, they allege, that the caufes which generally produce the difeafe, likewife create gaftric impurities, fuch as paffions, colds, errors in diet, violent hæmorrhages, fedentary life, a fudden relaxation of the abdomen after the delivery, &c.; and farther inftance the benefit which emetics and purgatives have afforded in this difeafe. But granting that all thefe caufes are capable of promoting the fecretion of bile and inteftinal tranfpiration, and accumulating fæces in the bowels; are we therefore to fuppofe, that they have no other effect upon the body? Why might they not immediately influence the fecretion of the milk, fince they never change the excitability of the body? Why may not they directly produce fpafms, without the medium of a fharp bile? Does not experience teach us how much and how quickly the quality of milk may be altered by vehement paffions, refrigerants, &c.? And in refpect to evacuating remedies, have thefe no other ufe than to remove a bilious acrimony and accumulation of fæces? And can we forget the power of emetics in increafing abforption? Do not purgatives operate in a manner fimilar to thefe? And is it not acknowledged, that they only prove falutary in this particular cafe, when milk is depofited within the bowels; and that in a metaftafis to the cavum abdominis, the breaft, or other parts, every violent diarrhœa is of a prejudical tendency?

I have always obferved an obftructed fecretion and metaftafis of the milk to any part of the body, as an effential fymptom of this difeafe; and the danger attending it depends on the fpot where the milk has been depofited; all other phenomena obfervable during the difeafe or death of the patient being merely accidental, as inflammations of the bowels, of the uterus, and the adjacent parts, gaftric complaints, &c. which fymptoms are eafily to be diftinguifhed from thofe attending the real puerperal fever. Induced by thofe circumftances, I am inclined to think, that the character of this difeafe originates "in the milky matter being contained in the blood in a fuperabundant quantity, which is occafioned either by an obftructed fecretion in the breafts, or by its being abforbed, and thus mixed with the mafs of blood, whence it is depofited in an heterogeneous part of the body." Such a confiderable quantity of milky matter retained in the blood, occafions by its ftimulus febrile motions, the vehemence of which depends on the greater or lefs quantity of milk that is accumulated in the blood; and it appears on the whole, that they become lefs violent whenever a part of the milky matter is depofed in the abdomen or any other part of the body, and that they increafe again at every new accumulation of milk in the blood. Milk feems to excite irregular febrile motions by ftimulating the veffels like any other heterogeneous matter brought into the blood; a circumftance that is evidently proved by the febris lactea, which is the more violent the more the excretion of milk from the breaft is impeded, and which confiderably diminifhes and ceafes entirely as foon as the child is fuffered to fuck. The febrile motions and other complaints, which are generally obferved at the period of weaning, can likewife not be accounted for, but from the milk ftimulating the veffels. In the puerperal fever, however, the body is in general debilitated, and by the topical fymptoms attending it, it appears to differ from the common febris lactea.

There can be no doubt that this fever varies from the febrile motions of what are called gaftric or inflammatory difeafes, efpecially in refpect of its uncertain exacerbations, and the frequent returns of chillinefs; moreover, on account of the great inequality of the pulfe, which at one time is full and hard, at another foft; fometimes fcarcely perceptible and intermittent, but always very frequent. It therefore cannot be fuppofed, that any one who has obferved feveral puerperal fevers fhould ever be led to miftake it for a common gaftric or inflammatory fever; or, in other words, for a fynochus or afthenic fever with impurities of the primæ viæ, or for a fthenic fever. This difference is ftill more ftriking when other difeafes of a fthenic or afthenic nature, for inftance, violent inflammations of the uterus, of the bowels, &c. precede the puerperal fever, a circumftance which I have often had an opportunity of obferving, as the inflammations of the bowels of the lower belly frequently occafion puerperal fevers. It is on this ground that fome found their opinion of the puerperal fever being nothing elfe but an inflammation of the uterus or inteftines, which error will be obviated by an accurate obfervation of any fuch cafe, case. Allowing the puerperal fever to be a common gaftric or fthenic fever, with a local inflammation, how are we to account for thofe extraordinary fymptoms with which that difeafe is attended; namely, the fwelling of the belly, impeded refpiration, violent pains in the belly or in the breaft, the fwelling of individual limbs, particularly the legs, the milky expectorations and excretion, the four fmell of breath and fweat? for hitherto I have not met with any puerperal fever without thefe fymptoms. If it be faid, that thefe indeed are confequences of lacteal metaftafes, but merely accidental, and only connected with inflammatory or gaftric fever, fo far as this occafions lacteal metaftafes; it appears to me, that in fome cafes there may be reafon for this affertion, fince it is certain, that lacteal metaftafes and puerperal fever, occafionally, though not always, follow after an inflammation of the genitals and dietetical aberrations. But if an inflammation be fuppofed by fome always to attend every puerperal fever, and by others a gaftric ftimulus be confidered as a conftant attendant, experience will fhow, in oppofition to both, that frequently there neither is any trace or veftige of the one nor of the other, although every effential fymptom of puerperal fever fhould be prefent.

On thefe grounds I am perfuaded, after the obfervations which I hitherto have made, that the puerperal fever is nothing but a morbid accumulation of the milky matter in the blood, and a depofition of the fame in fome particular part. That milky matter accumulated in the blood, operates indeed as a ftrong ftimulus on the fyftem, and might, under other circumftances, produce a fthenic difeafe; but here it fhews its effect upon a body debilitated by the preceding powers, and in general upon a habit of accumulated excitability, and confequently will but rarely occafion pure *sthenicæ*. And even if this fhould feem to be the cafe in fome inftances, by which a phyfician might be mifled to take this diforder for fthenia, it happens that very fpeedily a hyperfthenia makes its appearance; and by a diminution of the ftimulus you would weaken the body too much to obtain ftrength enough for the reforption of the milk depofited in improper parts, and for a juft fecretion of the fame towards the breafts.

The anomalous depofition of the milk may be very infignificant, and the greateft part of milky matter may remain in the blood; or, on the other hand, that depofition may alfo be confiderable, and the blood difcharge the greateft part of the milky fubftance contained in it. In the firft cafe the fever is always very violent, and the diforder will for a long time remain doubtful. But as foon as a great portion of the milky matter is conveyed to the belly or to the breaft, the violence of the fever abates, till the milky matter is again accumulated; at the ſame time, however, from the effuſion of the milk, certain ſymptoms will take place, which the more critical they are, the more eſſential that part is for the preſervation of life; and the more difficult it is to remove that matter, the more conſiderable the quantity in which it has been depoſited. The malady is always attended with leaſt danger when the milky matter is lodged in ſome limb which ſwells, and having broken, evacuates a ſubſtance reſembling pus or milk, according as the abſceſs is burſt ſooner or later. The cure may be protracted on account of that local complaint, but I never have ſeen any caſe of this kind which proved fatal. It is far worſe, when, without a return of the milk to the breaſt, or a diſcharge of it by ſtool or urine, the limbs, particularly the lower ones, ſwell in an œdematous manner, when theſe neither ſhow elaſticity, and when the tumour either diſappears quickly or conſtantly increaſes. I have had a caſe of this nature, which, with increaſed ſwelling, became fatal.

When the milk ſettles in the belly, it is more dangerous, if it be in the abdomen (which is by far the moſt common caſe, and has, therefore, more particularly been denominated puerperal fever) than if it be carried into the inteſtines, the uterus, or the vagina. The former caſe, however, is not always fatal when properly treated; and in proof of this aſſertion I ſhall, at the end of this Treatiſe, ſubjoin the notice of ſeveral inſtances in which, according to concluſions formed from continued pains in the belly and the ſwelled abdomen, the patient was thus affected. But thoſe ſymptoms, which are excited by a great quantity of milk being poured into the abdomen, when it often becomes ſour and caſeous, are always painful and alarming.

It is alſo eaſily to be conceived, that matter ſo foreign to theſe parts muſt produce a ſtrong ſtimulus, and beſides local affection and inflammation, excite a violent fever, altogether rendering the whole courſe of the diſorder very irregular. If the patient eſcape with her life, it is difficult to remove that matter by reſorption, when it once has been incruſted round the inteſtines; and to judge from ſeveral diſſections which have been inſtituted after unſucceſsful cures, a concreſcence of the bowels and other inteſtines, which naturally would create many inconveniencies, ſuch as barrenneſs, want of digeſtion, &c. is inevitable. The danger is ſtill farther increaſed, and the general health more injured, when the milky ſubſtance is depoſited on the lungs and in the thorax. Of the firſt caſe I have ſeen more than one inſtance, in which perfect health was apparently reſtored, though it is to be ſuppoſed, that at the ſame time time milky matter was lodged in the cavity of the thorax; but notwithftanding thefe favourable appearances, a fpeedy death or an afthma, occafioned by an obduration or adhefion of the lungs, or a difpofition to pulmonary ulcers, which are difficult to remove and frequently terminate in death, are the common confequences.

But lately a woman applied to me, who after an incautious ftoppage of the milk, retained a fhort breath and little cough; and not long ago a lady of my acquaintance died of a pulmonary ulcer, which had originated in a ceffation of the milk and of the lochiae, in confequence of a cold caught before the time of delivery, although no lacteal metaftafis towards the lungs was at firft apparent. If any confiderable portion of milk be thrown into the cavity of the thorax itfelf, in all probability death muft take place under fymptoms of fuffocation, in the fame way as in pulmonary inflammations, in which too much coagulable lymph penetrates into thefe parts.

In one cafe where this feemed to be the fact, though I cannot be quite certain, on account of diffection being denied, the patient died under the fymptoms alluded to.

The exciting caufes are in moft inftances colds, and that difpofition of the air which produces catarrhal fevers. Whenever I obferved feveral patients labouring under the puerperal fever at the fame time, I always found that a catarrhal conftitution of the atmofphere predominated, which has likewife been noticed by Vogler, (vid. *Loder's Journal for Surgery*, &c. in German) and others; and in that patient, with whom I was laft engaged, a common catarrhal fever preceded, from which fhe was almoft recovered, when the puerperal fever fhowed itfelf, which as the woman was in the country, was fuffered to go on for feveral days before my affiftance was called in. Often grofs errors in diet, perturbations of the mind, and an inconfiderable repulfion of the milk, were the caufes of this diforder.

Another caufe, not lefs frequent, is a difficult delivery, by which the internal or external parts of generation are injured, and an inflammation and gangrene occafioned. Sometimes purging medicines improperly given, though they might be gentle, if they were not blended with corroborants, feemed either to produce or at leaft to accelerate the lacteal metaftafes. At other times, violent hæmorrhages and fpafmodic pains of labour, which bore teftimony to a debilitated fyftem, were the forerunners of the diforder. I never obferved any gaftric fever as antecedent, though I am of opinion, that many a phyfician would have treated a preceding fever of that kind in an antigaftric manner. I have not hitherto made ufe of emetics, though I am inclined to believe, that fometimes they may be applied applied with advantage, even without the purpofe of evacuation. Upon the whole, the exciting caufes which have fallen under my obfervation were all debilitating: For I fhould not venture to affert of any one, adduced before, that it had been purely fthenic, as the condition of a perfon in child-bed, who has undergone the fufferings of a long and painful delivery, cannot well be confidered as fthenic.

It generally happens, that the phyfician is confulted when it is too late to obviate the firft operation of the caufes by proper remedies, and he meets with a diforder, which being partly local, and partly general, will give way to no fimple method of cure, either roborant or debilitating. If he is fortunate enough to fee the patients in the beginning, it is incumbent upon him to accommodate his mode of treatment to the particular fituation of the perfon; and having fucceeded in removing the diforder, to ufe all poffible diligence in checking the approach of puerperal fever, nor lofe a moment of his time in vain. According to the notions before explained, the endeavour of the phyfician, in the cure of puerperal fever, fhould be directed to prevent an unnatural tranflation of the milk to other parts, to conduct the milk again towards the breafts, and to remove the morbid matter which has been fecreted, either by reforption or in any other way. Moft caufes which produce puerperal fevers, manifeftly have a debilitating influence on the body, but it would be a great mifnomer to treat this malady according to the principles of the Brunonian theory, like any other typhus. Without entering into a ufelefs difcuffion of this theory, in order to fhow how much depends in the different modifications of afthenic difeafes upon the ufe of fome ftimulants, I will only remark, that the advocates of that theory have it not always in their power to abide by their principles, but are obliged in different afthenic cafes to prefcribe alfo fuch remedies, the benefit of which experience has taught us. We therefore find in their writings, in cafe of dropfy, that befides the roborants, fuch remedies are propofed, which always have been known as diuretics, though according to the theory of excitement, they ought to be confidered as unneceffary.

Guided by my own obfervations, I deem myfelf juftified in rejecting the indifcriminate ufe of ftimulants, and confining myfelf only to fome, though upon the whole a ftimulating method is preferable to a debilitating cure, becaufe the latter, according to experience, is apt to divert the milk from the breafts. The remedies of which I have chiefly availed myfelf in this diforder, are to be reckoned among the ftimulants, and to that particular clafs of them which are commonly called anti-fpafmodic. They are valerian and opium; the former in a greater dofe than I have feen prefcribed by many phyficians. Thefe medicines, medicines, of which I apply the opium only in ſmall quantities, that is to ſay, from half a grain down to a quarter of a grain, and perhaps even in ſtill ſmaller portions, to be given every hour or every two hours, prove to be the moſt ſuitable for the purpoſe of producing anti-ſpaſmodic effects, and for diminiſhing the fever, by reſtoring a certain degree of order in the proceſs of lacteal ſecretion. However, with ſome mixture, theſe remedies, even when I preſcribed opium in very trifling doſes, ſtill ſeemed too ſtimulant, and I was frequently obliged to omit the latter entirely. To be able to apply it in ſtronger doſes, eſpecially in a troubleſome diarrhœa, I generally added alkali, ſaturated with vinegar, which addition diminiſhes the power of opium. It alſo has the advantage of promoting the reſorption of extravaſated milk, and the diſcharge by the urine, which is a more proper way of evacuation, in this inſtance, than the bowels or the lungs.

[ To be continued. ]

